# Choroid Plexus Aquaporins in CSF Homeostasis and the Glymphatic System: Their Relevance for Alzheimer’s Disease

**DOI:** 10.3390/ijms24010878

**Published:** 2023-01-03

**Authors:** Cristina Municio, Laura Carrero, Desireé Antequera, Eva Carro

**Affiliations:** 1 Group of Neurodegenerative Diseases, Hospital Universitario 12 de Octubre Research Institute (imas12), 28041 Madrid, Spain; 2 Network Center for Biomedical Research in Neurodegenerative Diseases (CIBERNED), ISCIII, 28031 Madrid, Spain; 3 Neurobiology of Alzheimer’s Disease Unit, Functional Unit for Research into Chronic Diseases, Instituto de Salud Carlos III, 28222 Madrid, Spain

**Keywords:** aquaporins, choroid plexus, cerebrospinal fluid, glymphatic system, Alzheimer’s disease, astrocytes, clearance, homeostasis

## Abstract

The glymphatic system, a fluid-clearance pathway involved in brain waste clearance, is known to be impaired in neurological disorders, including Alzheimer’s disease (AD). For this reason, it is important to understand the specific mechanisms and factors controlling glymphatic function. This pathway enables the flow of cerebrospinal fluid (CSF) into the brain and subsequently the brain interstitium, supported by aquaporins (AQPs). Continuous CSF transport through the brain parenchyma is critical for the effective transport and drainage of waste solutes, such as toxic proteins, through the glymphatic system. However, a balance between CSF production and secretion from the choroid plexus, through AQP regulation, is also needed. Thus, any condition that affects CSF homeostasis will also interfere with effective waste removal through the clearance glymphatic pathway and the subsequent processes of neurodegeneration. In this review, we highlight the role of AQPs in the choroid plexus in the modulation of CSF homeostasis and, consequently, the glymphatic clearance pathway, with a special focus on AD.

## 1. Introduction

Protein aggregates are a common feature of neurodegenerative diseases, including Alzheimer’s disease (AD) [1]. This implies that reduced brain clearance, which results in the accumulation of anomalous proteins, could be a shared phenomenon in neurodegeneration. Cerebrospinal fluid (CSF), the main component of the extracellular fluid in the central nervous system (CNS), is a vital pathway for waste clearance from the neural tissue [2,3]. Analytical studies of CSF molecules have revealed that they can act as biomarkers for the diagnosis of clearance failure from the CNS in diseases such as AD.

Along perivascular spaces, CSF transport partly involves the metabolic waste disposal pathway in the brain known as the “glymphatic system” [4]. The glymphatic system works as a waste drainage pathway comprising a perivascular network for CSF transport [4,5], and is connected to the lymphatic system, associated with dura covering the brain as well as cranial nerves and large vessels at the skull exits [6,7]. The processes of these structures are intricate and only partly understood.

In the glymphatic pathway, a convective CSF influx is compensated by the perivenous efflux of the interstitial fluid (ISF), clearing noxious proteins and peptides, including amyloid-beta (Aβ) [4]. CSF movement into the parenchyma, facilitated by aquaporins (AQPs), drives the convective ISF fluxes within the tissue toward the perivenous spaces [4]. The consequent CSF transport into the brain parenchyma is mainly facilitated by AQP4 water channels, which are expressed in a polarized way in the astrocytic end-feet surrounding the brain vasculature [4,8].

In the last decade, several studies reported that soluble Aβ protein and tau oligomers are removed from the brain through the glymphatic system [4,9,10,11,12]. These emerging findings prove promising for the development of novel therapeutic targets for the prevention of AD pathogenesis. In this review, we address recent findings on CSF and the glymphatic system, with a special interest in the role of the choroid plexus and AQP expression, to provide insights into the mechanisms underlying this complex clearance system.

## 2. Choroid Plexus

**Figure 1:**
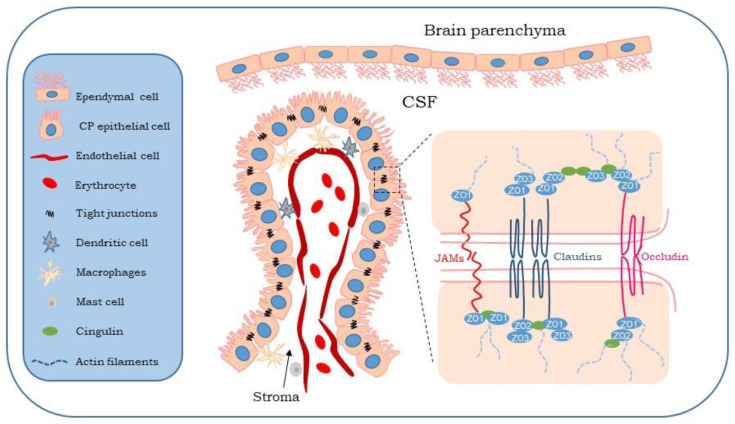
Illustration of choroid plexus epithelial cells (**left**) and tight junctions at the luminal membrane (**right**). CP: choroid plexus; CSF: cerebrospinal fluid; JAMs: junctional adhesion molecules; ZO: zonula occludens.

To maintain the extracellular environment of the brain, the blood–brain barrier (BBB) and the blood–CSF barrier (BCSFB) separate the blood from the brain parenchyma and the CSF, respectively [13]. The BCSFB is primarily composed of the choroid plexus epithelial cells that are connected by tight junctions restricting access to the brain parenchyma [14] (Figure 1). The remainder of the BCSFB is composed of the arachnoid membrane and circumventricular organs. The mammalian choroid plexus is a highly vascularized secretory tissue localized in the brain ventricles [15]. The choroid plexus tissue includes a monolayer of epithelial cells surrounding the connective tissue with fenestrated capillaries inside. The apical membrane of the choroid plexus epithelium is covered by microvilli, which increase the membrane area in contact with the intraventricular space. Microvilli primarily consist of cilia and motile cilia, with sensory and motile functions, respectively [16].

The choroid plexus epithelial cells are sealed by tight junctions at the luminal membrane conferring the barrier property to the BCSFB. Tight junctions are composed of proteins associated with the cell membrane of the choroid plexus epithelial cells [17,18,19]. Occludin was the first tight junction protein to be reported in the choroid plexus [20]. Another group of tight junction proteins is claudins, divided into several subtypes, among which claudin 1–6, 9–12, 19, and 22 are expressed in the choroid plexus epithelial cells [21], limiting the through-put of tight junction [22]. Zonula occludens (ZO) protein is another tight junction protein in the cuboidal choroid plexus epithelial cells [23]. Three ZO proteins, namely ZO1, ZO2, and ZO3, bind occludin and claudin to actin filaments [24]. Junctional adhesion molecules (JAMs) are tight junction proteins that have been implicated in the epithelial barrier function [23] (Figure 1). They have short cytoplasmic tails to bind to proteins such as ZO1, cingulin, and occludin, which allows the formation of multiprotein complexes with tight junctions [25,26].

The major function of the choroid plexus is to form the BCSFB and produce the CSF. For this purpose, the plasma that flows through the fenestrated capillaries is rapidly transported by hydrostatic pressure into the choroid plexus interstitium. Once filtered by the membranous diaphragms, the ultrafiltrate is transported through the choroid plexus epithelial cells to the ventricle, where it can be used in CSF production [27].

Contrary to other secretory epithelia, in the choroid plexus epithelium, most ion transporters are located in the apical membrane, for example, Na+-K+ ATPase channels, K+-Cl-Na+HCO3 cotransporters, or some AQP water channels. Only a few transporters, such as K+Cl- cotransporters, are found on the basolateral membrane [28]. In addition, in recent decades, other functions have been associated with the choroid plexus such as the modulation of CSF composition, CNS development, and regeneration, the removal of waste and metabolites, brain immunosurveillance, or circadian rhythmicity regulation [29,30,31,32,33,34,35,36].

Several significant alterations have been indicated in the aging choroid plexus. A loss of 11% in the volume of human choroid plexus epithelial cells has been associated with aging. In addition to collagen fibers and dystrophic calcifications within the stroma, aged choroid plexus epithelial cells show increased pathological protein deposits called Biondi ring tangles [37,38]. Reduced glucose metabolism and energy production have been observed in the choroid plexus of aging rats [39]. Furthermore, the accumulation of toxic products and lipofuscin, a product of lipid peroxidation, indicates higher oxidative stress in the aging choroid plexus [40].

Morphological changes in the choroid plexus have been described in AD, including the atrophy of choroid plexus epithelial cells with the accumulation of lipofuscin vacuoles, stromal fibrosis, and the thickening of blood vessel walls and the basement membrane of the choroid plexus [41,42,43,44]. Larger choroid plexus volume was associated with the severity of cognitive impairment in the AD spectrum [45]. These structural and molecular alterations in the choroid plexus lead to impairment of the BCSFB that may result in changes in CSF composition in the AD brain [13,46,47,48,49]. Increasing lines of evidence suggest that choroid plexus dysfunction could be a major factor contributing to AD pathogenesis [46,50,51]. Aβ accumulation in the choroid plexus affects its structure and function. We and others demonstrated that Aβ deposition in the choroid plexus of AD patients results in impaired physiological functions of the choroid plexus epithelium, such as a decrease in mitochondrial activity, oxidative stress, and morphological/structural alterations [46,52,53,54,55,56].

Aβ presence in the choroid plexus may induce innate immune reactions, increasing the concentration of IgG that leads to capillary damage and interstitial fibrosis [57]. It has previously been reported that the expression of type I and II interferons, involved in the recruitment of immune cells to the CNS, is altered in the choroid plexus in AD [58]. In the early stages of AD, the presence of Aβ can increase the secretion of proinflammatory cytokines (IL-1, IL-6, and TNF-α) and matrix metalloproteinases, leading to the downregulation of tight junction proteins and thus BCSFB dysfunction [46].

It is well known that the primary role of the BCSFB is to prevent the free passage of molecules between the blood and the CSF, and the main function of the choroid plexus is to the production and regulation of the CSF [2,14,27,59]. However, this specialized tissue is also involved in neuroimmune surveillance, as immune cell exchange between the blood and the CSF occurs at the choroid plexus level [34,35,36]. The choroid plexus manages immune cell recruitment into the CNS under several pathological situations, including AD [60]. Many studies have shown an increase in the number of immune cells in the choroid plexus and the CSF following immune challenges to the CNS [61,62,63,64]. A variety of immune cells, including macrophages, basophils, mast cells, dendritic cells, monocytes, neutrophils, and lymphocytes, are present in the choroid plexus [65]. However, choroid plexus macrophages are the most abundant immune cells [64]. Notably, the immunological network preserves the immune surveillance of the CNS from outside the parenchyma, and under pathological conditions, it contributes to neuroinflammation. This reaction is believed to be mediated by the choroid plexus, which serves as a selective gateway for leukocyte entry [34].

Inflammation is an important defensive response to infection or injury, and the choroid plexus is known as an entry site for pathogens, a checkpoint for peripheral immune cells into the CNS, and a regulator of cytokines and other signaling molecules in the CSF [58,66,67]. Specifically, TNF-α signaling is induced in the choroid plexus of AD patients and AD mouse models [68].

Additionally, the choroid plexus is involved in the control of circadian rhythmicity [31,32,33]. The behavioral circadian rhythm is an integrated output of multiple brain clocks, for which the choroid plexus is an essential element. Indeed, the presence of a circadian clock in the choroid plexus has been associated with a robust circadian gene expression rhythm [31]. The circadian rhythmicity of choroid plexus clock genes differs with sex, exhibiting more diurnal differences in female rats than in male rats [69]. It is established that estradiol impacts the expression of clock genes, modulated by the estrogen receptor [69]. More recently, we revealed the dysregulation of circadian rhythmicity in the choroid plexus of a mouse model with AD [70].

## 3. Glymphatic System

**Figure 2:**
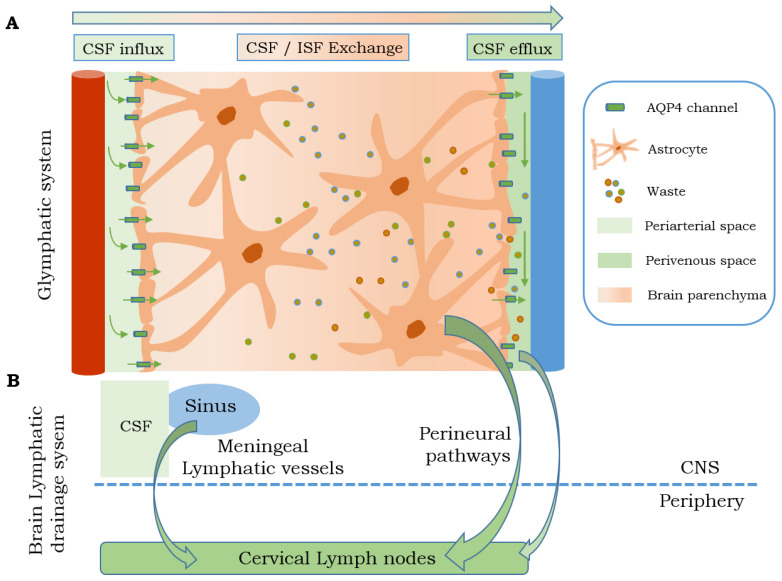
Brain lymphatic drainage system. (**A**) Glymphatic system clears solutes and waste of the brain parenchyma due to the influx of CSF from the periarterial space. Once the CSF is mixed with the ISF, they drain into the perivenous space. Part (**B**) shows a summary of the other brain drainage systems that transport the CSF through the meningeal lymphatic vessels and/or perineural pathways, such as olfactory nerve, to the cervical lymph nodes AQP: aquaporin; CNS: central nervous system; CSF: cerebrospinal fluid; ISF: interstitial fluid.

In 2012, Nedergaard and colleagues defined the glymphatic system as a network of perivascular spaces through which the CSF circulates in the brain parenchyma [4]. The glymphatic system involves the entry of subarachnoid CSF into the brain parenchyma through periarterial spaces, after which it mixes with parenchymal ISF and interstitial waste products, facilitated by the AQP4 water channels located in astrocytic end-feet, and drains through the perivenous spaces surrounding the veins (Figure 2) [4]. The glymphatic pathway is a highly organized fluid transport system and has a three-step serial process. First, the CSF flows from the subarachnoid space into the brain through the perivascular spaces of large leptomeningeal arteries; then, it is driven into the brain parenchyma through the perivascular spaces of the penetrating arteries. This movement is facilitated by the positive CSF pressure from the choroid plexus and the arterial pulse caused by the cardiac cycle. Afterward, the CSF flows across the basal membrane and astrocytic end-feet, bordering the brain parenchyma, where AQP4 contributes to the CSF flow into the parenchyma, mixing with the ISF [4,7,71]. The CSF and ISF are in continuous exchange, and both are cleared together with solutes. The flux passes from the interstitium into specific perivenous pathways and is drained through a meningeal lymphatic vessel, thus reaching the cervical lymphatic system [4,72].

It is well documented that the CSF and the cerebral ISF drain into deep cervical lymph nodes, but the CSF also drains into the perineural space. In fact, perineural pathways along the optic and olfactory nerves are considered relevant lymphatic routes [73,74]. However, it remains unknown whether waste solutes are mainly cleared through perineural drainage, thus traveling along the cranial nerves, or drain into the nerve itself. Although more studies are needed, it has been reported that the CSF and waste solutes predominantly drain directly into lymphatic vessels along the exiting cranial nerves and not directly into the venous circulation [7].

Most neurodegenerative diseases are characterized by the improper accumulation of cellular waste products, among which misfolded proteins are the most difficult to clear from the brain, and their aggregates lead to diseases such as AD [5]. The glymphatic system has been recently discovered as an alternative waste clearance system enabling the removal of soluble proteins and metabolites from the CNS [4,71]. The designation of the glymphatic clearance pathway refers to its appropriation of the lymphatic function for interstitial protein management and its dependence on glial water transport [75].

Although the best-described function of the glymphatic system is solute clearance, studies are underway on other possible functions such as facilitating the transport of glucose [76], apolipoprotein E (ApoE) [77] or lipids [78]. The BBB restricts the influx of lipids and lipoproteins into the brain. Internal lipid transport occurs through the secretion of lipoproteins by astrocytes, mainly apolipoproteins. These carriers are central for the preservation of brain homeostasis, as they mediate the clearance of excess cholesterol and Aβ, and their deficiency is a risk factor for AD [79]. Part of the ApoE production occurs in the choroid plexus of the third ventricle, which is directly secreted into the CSF [77]. Therefore, glymphatic transport pathways contribute to the distribution of ApoE into the brain [77].

Glymphatic activity decreases with advanced age. Studies in mice show a reduction in the efficiency of clearance and loss of perivascular APQ4 polarization in the astrocytes of aging mice compared with young mice [80]. AQP4 is localized in the astrocytic end-feet of young animals; however, the polarization of these water channels is reduced in aging reactive astrocytes, characterized by reactive gliosis [80]. With advanced age, CSF production decreases, along with the stiffening of the arterial walls, leading to a reduction in glymphatic activity [81].

It has been established that the glymphatic system is more effective during sleep and is largely inactive during wakefulness [11,82,83]. During sleep, the brain’s extracellular space is enlarged, thus reducing resistance to fluid flow and promoting more CSF infiltration in perivascular spaces [84,85]. Based on these findings, the sleep cycle enables more effective CSF clearance in the glymphatic system by eliminating the metabolic waste generated during wakefulness. Thus, interruptions in the sleep cycle would functionally impair glymphatic clearance, resulting in defective waste removal and the potential accumulation of neurotoxic elements such as Aβ [11,86,87,88]. This finding is in line with the previously reported findings showing that sleep deficiency significantly raises Aβ levels, thus increasing its deposition [89]. Coincidentally, most AD patients suffer from circadian rhythm disorders [90,91,92]. Therefore, improvement in the sleep quality of AD patients could be a potential therapeutic approach by helping recovery or at least preventing pathological evolution.

The glymphatic system is one of the numerous mechanisms that contribute to the removal of soluble Aβ from the brain [93]. AD patients display altered CSF dynamics, potentially causing an imbalance in the production and clearance of soluble Aβ, resulting in the accumulation of Aβ in the brain [94]. Additionally, cerebral Aβ deposition was associated with arterial stiffness, which contributes to a disruption of vascular dynamics and complicates the perivascular flow of Aβ [95]. This results in a detrimental cycle in which Aβ deposition along the blood vessels impairs the glymphatic function and promotes more severe Aβ accumulation in the parenchyma and ultimately neuronal death.

AQP4, located on the perivascular astrocytic end-feet, enables the exchange of CSF and ISF and facilitates CSF influx into the brain parenchyma and its efflux back to the perivascular space [96]. Owing to the findings of Iliff and colleagues, who revealed a decrease in CSF influx and glymphatic function due to AQP4 knockout, it is now established that the functionality of the glymphatic system highly relies on AQP channels [4]. Nevertheless, several of the key structural and functional aspects of the glymphatic system are still debated [97], including whether it involves a convective flow or a passive diffusion process [86]. However, AQP4 is not the only factor involved in the glymphatic system; AQP1 also has a significant role in ISF and CSF homeostasis [98,99,100].

## 4. Aquaporins

AQPs are a heterogeneous group of channel-forming proteins found in all kingdoms of life [101]. AQPs are small transmembrane proteins that mediate the transport of water and some small non-charged molecules, such as glycerol, sugars, or gases, through the plasma membrane. This process is driven by solute and osmotic gradients [102]. To date, 13 types of AQPs (AQP0-12) have been identified that, besides having permeability features, enable specific subcellular and tissue localization, which suggests an association between the function and site of expression [103]. They are divided into two main categories, classical and non-classical. Classical AQPs, namely AQP0, AQP1, AQP2, AQP4, AQP5, AQP6, and AQP8, are involved in water permeability. This group also includes aquaglyceroporins, i.e., AQP3, AQP7, AQP9, and AQP10, which support the transport of some neutral solutes and glycerol, in addition to water. Superaquaporins, or non-classical AQPs, include AQP11 and AQP12. The role of these AQPs has not yet been identified since their physiological function contributing to water permeability is not yet clarified [104].

AQPs are tetramers and comprise monomers with identically hydrophilic membrane proteins. Each monomer includes six transmembrane alpha helix segments and two short helix segments, joined by five connecting loops. The carboxyl and terminal amino domains are located on the intracellular side. This distribution allows AQPs to operate as a hydrophilic integral membrane protein [105]. The central pore is composed of two consensus motifs of asparagine–proline–alanine (NPA) in the short segment. The presence of AQPs in biological membranes is regulated by exocytosis and endocytosis [106].

The distribution of AQPs includes different organs and tissues, which have been associated with a variety of important physiological functions including brain water homeostasis, transepithelial fluid transport, osmoregulation, angiogenesis, wound healing, cell signaling, migration, and proliferation [107,108]. In addition, AQPs have been suggested as potential targets for drug development [103].

In the brain, AQPs mediate the movement of water between different fluid compartments, including the CSF, the ISF, and the blood [109,110]. As recently reported by Trillo-Contreras and colleagues, nine AQPs have been identified in different sites of the CNS: AQP1, AQP3, AQP4, AQP5, AQP6, AQP7, AQP8, AQP9, and AQP11 (Table 1) [111].

## 5. AQPs in Choroid Plexus and Their Role in Glymphatic System

The expression of AQPs has not been extensively studied in the choroid plexus; however, there is evidence that AQP1, AQP4, AQP5, AQP7, and AQP11 are expressed in the epithelial cells of the choroid plexus.

AQP1 was observed on the choroid plexus epithelial cells of mice, where it is located in the apical membrane at the ventricular-facing surface of the choroid plexus epithelium [112,113,114,115,116,117,118,127,140,141]. The choroid plexus of aging rats showed lower levels of AQP1 expression, with decreased secretory activity [14]. Moreover, AQP1 expression was also characterized in the primary human plexus epithelial cells [119,120,121]. The main site of AQP1 expression is in the apical membrane domain, although in some cells, a low basolateral expression has also been detected [128]. AQP1 is a cGMP-gated cation channel [142,143], and functions as a water channel and a gated ion channel in the choroid plexus, contributing to the regulation of CSF production [98,109,144,145]. Based on experimental findings using AQP1 null mice, it is estimated that AQP1 participates in CSF production, generating between 20% and 25% CSF [115]. Importantly, in an AD triple transgenic mouse model, AQP1 expression was found to be reduced in the choroid plexus epithelial cells in parallel with reduced CSF production [146]. However, no significant change in AQP1 expression was observed in the choroid plexus of AD patients [48]. Therefore, more investigations are needed on human choroid plexus tissue, and age and disease comparisons should be further investigated. However, the potential impact of AQP1 on CSF/ISF homeostasis, including CSF production and drainage, CSF/ISF exchange, and the consequent CSF-mediated clearance systems, is unquestionable.

In the CNS, the most frequent aquaporin is AQP4. It is usually expressed in ependymal-glial-limiting membranes, including the ependymal cells and subependymal glia that border the brain ventricles, in the subpial astrocytes of the cortex, the end-feet of the perivascular astrocytes that surround the blood vessels forming the BBB, and the choroid plexus. The presence of AQP4 in the choroid plexus of rats was reported in some previous studies, in which they showed weak and diffuse AQP4 signals [122,126]. However, AQP4 overexpression in the choroid plexus is markedly promoted by hypoxia, contributing to the increase in CSF production observed under hypoxic events [140], but also by hypertension [127].

AQP4-positive cells were found in the choroid plexus of human donors, where the AQP4 signal was mostly observed in the basolateral membrane domain [128]. In this recent study, AQP4 mRNA levels were also present and increased in older mice, which suggests an association between advanced age and increased AQP4 expression, and therefore it could act as a compensatory mechanism to preserve CSF levels and reduce its production [128].

In a previous study by Iliff et al., it was found that AQP4, expressed in astrocytic end-feet in contact with the brain vasculature, facilitates CSF transport into the brain parenchyma [4]. The authors showed a drastic reduction in both CSF fluid flux through the mouse parenchyma and the clearance of intrastriatal-injected radio-labeled Aβ by genetic ablation of AQP4 in astrocytes [4]. Therefore, the perivascular glymphatic pathway, driven by AQP4-dependent bulk flow, has been proposed as the main clearance pathway of interstitial fluid solutes from the brain parenchyma [8].

Although the dependence of the glymphatic system on AQP4 has been demonstrated and reported in some studies [147], the physiological mechanism by which AQP4 at astrocyte end-feet facilitates glymphatic flux is not yet completely understood. Subcellular relocalization of AQP4, from intracellular vesicles to the plasma membrane, is critical in the modulation of AQP4 function, being a dynamic process independent of changes in AQP4 expression [148,149,150]. AQP4 is mainly found on astrocytic end-feet, allowing its contact with the perivascular space adjacent to the blood vessels, facilitating CSF influx and its efflux back to the perivascular space. AQP4 connects astrocyte cytoplasm with the ISF, allowing a dynamic fluid that facilitates interstitial movement, essential for the glymphatic flow [4]. Since AQP4 is also localized in choroid plexus epithelial cells as the basolateral level [128], AQP4 is implicated in other aspects of CSF homeostasis. It has been suggested that the choroid plexus epithelial cells express AQP4 to modulate CSF production, generating a higher transcellular waterflow that leads to normal waterflow and CSF production [128]. It was reported that CSF distribution is under circadian control and that AQP4 supports this circadian rhythm [151]. Thus, the expression and functionality of AQP4 in the choroid plexus are directly essential for maintaining regular CSF production and the consequent glymphatic activity. Alterations in any of the components, including AQP4, of this multifactorial pathway, may lead to an impaired glymphatic waste clearance function causing the accumulation of waste and neurotoxic proteins (e.g., Aβ, tau) which contribute to neurodegenerative diseases. Reduced perivascular AQP4 expression has been reported in the frontal cortical gray matter of subjects with AD compared to cognitively intact subjects. This AQP4 decrease was associated with increasing Aβ and neurofibrillary pathological burden, and with cognitive decline prior to dementia onset [152]. In addition to the well-described role of astrocytic AQP4, we believe that choroid plexus AQP4 expression may change with physiological and pathological conditions, with the following effects on glymphatic clearance activity that may contribute to the development of neurodegenerative diseases, including AD.

AQP5 has been also described in choroid plexus epithelial cells [118]. Moreover, with apical epithelial cell localization, AQP5 mRNA expression and protein levels were upregulated during posthemorrhagic ventricular dilatation [120]. Although AQP5 localization has been described in choroid plexus [118,120], its role on CSF homeostasis has not been demonstrated. However, its expression has been associated with brain edema [130]. A recent study has described the relationship between changes in AQP expression or distribution with ischemia, commonly associated with edema [111]. In other tissues such as salivary glands and the eye, AQP5 is involved in the production of primary saliva and tear formation, respectively, and their secretory regulation [153]. Other studies reported that AQP5, expressed in human salivary glands occasioned a significant rise in the osmotically directed net fluid [154], and AQP5 knockout results in reduced water secretion. AQP5 expression also influences the transepithelial water flux along the respiratory segments [155]. Thus, we suggest that AQP5 downregulation may lead to a reduction in CSF secretion seriously affecting the CSF/ISF homeostasis and the consequent glymphatic clearance system.

The aquaglyceroporin AQP7 mRNA and protein levels were largely found in the choroid plexus, increasing during perinatal development of the brain, and suggesting that AQP7 could be an important structural and functional element in the choroid plexus during brain development in mice [133]. Evidence of AQP7 in the apical membrane of the choroid plexus cells implies probable cooperation between AQP7 and AQP1 in CSF excretion [122,156]. However, these studies are not conclusive and these results would need to be confirmed.

AQP7 was first denoted as “aquaporin adipose” because it was initially described in human adipose tissue [157]. Next, other researchers found AQP7 expression in other tissue, including the choroid plexus [133,134]. Although the AQP7 role is mainly associated with fat metabolism, it is reported that APQ7 is directly involved in water transport in adipocytes [158]. AQP7-knockout mice were shown to develop reduced water permeability in the kidney [159]. Nevertheless, the role of AQP7 at the choroid plexus, as well as in the CSF secretion, requires further studies.

In rat choroid plexus a weak AQP11 expression has been reported [138]. More recently, AQP11 localization in the epithelium was confirmed in mouse brains [139]. At this time, the function of AQP11 at the BBB in water permeability is not well understood, thus its role in the choroid plexus remains unknown. AQP11 has been identified in intracellular organelles in different cell types, mainly in the endoplasmic reticulum [160]. Due to its intracellular location, its role in the transport of water and/or other solutes is still debated. Several studies have provided questionable data about AQP11 transport specificity [138,160], but other studies using in vivo and in vitro permeability assays, exposed that AQP11 may act as a functional water channel [161,162,163].

## 6. Conclusions

**Figure 3:**
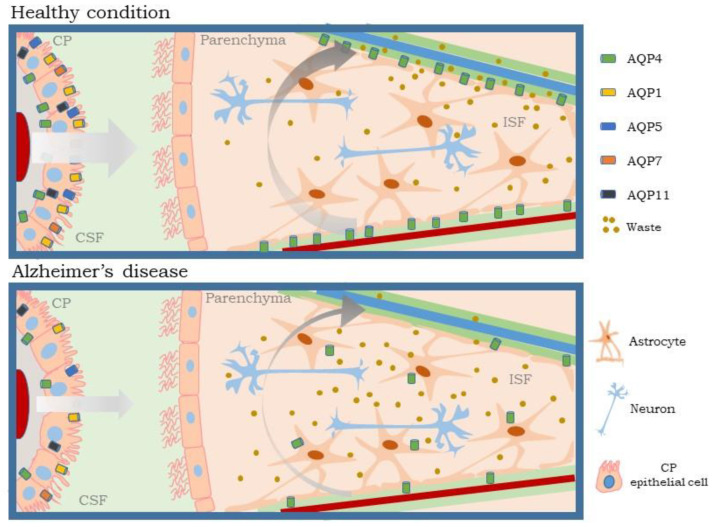
Relationship between choroid plexus and glymphatic system. In healthy conditions (**above**), the choroid plexus forms the BCSFB and produces CSF. CSF flows from the periarterial space to the brain parenchyma via AQP4, located in the end-feet of astrocytes. This movement is favored positive pressure of CSF production from the choroid plexus and the arterial pulse. The CSF/ISF mixture and waste products are cleared by passing into the perivenous space to be taken to the lymphatic tissues. In AD (**below**) the expression of AQPs decreases in choroid plexus epithelial cells causing a reduction in CSF production. The decrease in pressure exerted by the CSF together with the diminution and depolarization of AQP4 in the astrocyte end-feet, reduces the glymphatic function, preventing a correct clearance of the ISF and waste products. AQP: aquaporin; BCSFB: brain CSF barrier; CSF: cerebrospinal fluid; CP: choroid plexus; ISF: interstitial fluid.

Although the choroid plexus is involved in the production and regulation of one of the central components of the glymphatic system, the CSF, not enough attention has been paid to research on the glymphatic system. A balance between CSF production and drainage is required for operative waste removal through the clearance glymphatic pathway. In this environment, AQPs play a major role in CSF homeostasis. As we propose in Figure 3, impairments in choroid plexus functioning in AD, including AQPs expression and function, could directly disturb the glymphatic system’s efficiency, with the corresponding pernicious effects in the neuropathological processes driving AD.

The choroid plexus comprise an intricate network of complexly organized cells that, in addition to functioning as a barrier between the blood and CSF, have now been confirmed to play a major role in neurological processes, including AD. Currently, more understanding about the connection between the choroid plexus and glymphatic system is appearing, and in this review, we summarized some key elements to contribute highlight the role of AQPs in the choroid plexus modulating CSF homeostasis and, consequently, the clearance glymphatic pathway.

## Figures and Tables

**Table 1:** Distribution of aquaporins in brain’s tissues. Modified from Trillo et al., 2022 [111].

Protein	Tissue Expression	References
AQP1	Eye, choroid plexus, circumventricular organs, astrocytomas, sensory neurons of dorsal root, trigeminal and nodose ganglia	[112,113,114,115,116,117,118,119,120,121,122,123,124]
AQP3	Eye, astrocytes, neurons	[125]
AQP4	Subpial astrocyte end-feet, retina, neurons, circumventricular organs, hippocampus, ependymal cells, glial cells, Purkinje cells, choroid plexus	[122,126,127,128,129]
AQP5	Astrocytes, neurons, choroid plexus	[111,118,120,125,130,131]
AQP6	Cerebellum	[132]
AQP7	Brain development, choroid plexus	[133,134]
AQP8	Astrocytes, neurons, oligodendrocytes, astrocytomas	[125,135]
AQP9	Substantia nigra, tanycytes, astrocytes, spinal cord radial astrocytes	[131,134,136,137]
AQP11	Choroid plexus	[138,139]

## Data Availability

Data is contained within the article.
